# Bioresorbable optical sensor systems for monitoring of intracranial pressure and temperature

**DOI:** 10.1126/sciadv.aaw1899

**Published:** 2019-07-05

**Authors:** Jiho Shin, Zhonghe Liu, Wubin Bai, Yonghao Liu, Ying Yan, Yeguang Xue, Irawati Kandela, Maryam Pezhouh, Matthew R. MacEwan, Yonggang Huang, Wilson Z. Ray, Weidong Zhou, John A. Rogers

**Affiliations:** 1 Department of Chemical and Biomolecular Engineering, University of Illinois at Urbana-Champaign, Urbana, IL 61801, USA.; 2 Frederick Seitz Materials Research Laboratory, University of Illinois at Urbana-Champaign, Urbana, IL 61801, USA.; 3 Department of Electrical Engineering, University of Texas at Arlington, Arlington, TX 76019, USA.; 4 Department of Materials Science and Engineering, Northwestern University, Evanston, IL 60208, USA.; 5 Center for Bio-Integrated Electronics, Northwestern University, Evanston, IL 60208, USA.; 6 Department of Neurological Surgery, Washington University School of Medicine, St. Louis, MO 63110, USA.; 7 Departments of Mechanical Engineering and Civil and Environmental Engineering, Northwestern University, Evanston, IL 60208, USA.; 8 Developmental Therapeutics Core, Northwestern University, Evanston, IL 60208, USA.; 9 Northwestern Medicine, Feinberg School of Medicine, Northwestern University, Evanston, IL 60208, USA.; 10 Departments of Biomedical Engineering, Chemistry, Mechanical Engineering, Electrical Engineering and Computer Science, and Neurological Surgery, Simpson Querrey Institute for Nano/biotechnology, McCormick School of Engineering and Feinberg School of Medicine, Northwestern University, Evanston, IL 60208, USA.

## Abstract

Continuous measurements of pressure and temperature within the intracranial, intraocular, and intravascular spaces provide essential diagnostic information for the treatment of traumatic brain injury, glaucoma, and cardiovascular diseases, respectively. Optical sensors are attractive because of their inherent compatibility with magnetic resonance imaging (MRI). Existing implantable optical components use permanent, nonresorbable materials that must be surgically extracted after use. Bioresorbable alternatives, introduced here, bypass this requirement, thereby eliminating the costs and risks of surgeries. Here, millimeter-scale bioresorbable Fabry-Perot interferometers and two dimensional photonic crystal structures enable precise, continuous measurements of pressure and temperature. Combined mechanical and optical simulations reveal the fundamental sensing mechanisms. In vitro studies and histopathological evaluations quantify the measurement accuracies, operational lifetimes, and biocompatibility of these systems. In vivo demonstrations establish clinically relevant performance attributes. The materials, device designs, and fabrication approaches outlined here establish broad foundational capabilities for diverse classes of bioresorbable optical sensors.

## Teaser

Bioresorbable optical sensor implants monitor brain pressure and temperature before naturally resorbing into the body.

## INTRODUCTION

Implantable sensors capable of precise, continuous monitoring of pressure and temperature within organ spaces associated with the brain, heart, eyes, and bladder provide essential diagnostic information for defining treatment protocols for diseases such as traumatic brain injury, cardiovascular abnormalities, glaucoma, and neurogenic bladder dysfunction, respectively (*1*–*3*). Among various conventional sensor technologies, optical devices are finding increasing utility in these and other contexts due to their immunity to electromagnetic fields, thereby offering improved compatibility with clinical imaging techniques such as magnetic resonance imaging (MRI) (*4*–*6*). More specifically, the absence of conductive traces and electrically powered circuit components greatly reduces adverse events that can arise from Joule heating (*7*, *8*), dislocation (*9*), and image distortion (*10*) induced by electromagnetic interactions during the MRI, or current leakage that could follow from defects in the device encapsulation (*11*). The permanent nature of the constituent materials represents a disadvantage for all existing sensors, both electrical and optical, because of their need for surgical removal after a useful operating period. These procedures are costly and can lead to additional complications and risks to the patient (*12*). Such permanent devices can also serve as a nidus for infection (*13*, *14*), and they can lead to immune-mediated inflammatory responses.

Solutions to these challenges may follow from the use of materials, device designs, and fabrication strategies in emerging classes of bioresorbable electronic sensor systems. These types of devices can be constructed to dissolve harmlessly in biofluids at well-defined, programmable rates to biologically benign end products. Here, processes of bioresorption naturally eliminate the devices at their sites of implantation after relevant operational time frames, thereby bypassing the need for surgical extraction. Recent demonstrations include biophysical sensors of pressure, temperature, flow rate, and motion (*15*–*17*), several types of biochemical sensors (*15*, *18*), neural electrodes (*19*), and power supplies (*20*). Published in vivo studies include deployments in the intracranial and intra-abdominal spaces and in the leg cavities (*15*, *17*), on cortical surfaces (*19*, *21*), and in subdermal regions (*22*). Substituting basic components of these systems, such as the sensing element [silicon nanomembranes (Si NMs)], device architectures (microelectromechanical or thin-film designs), and electrical interconnections (bioresorbable metal electrodes), with optical analogs offers the potential to establish routes to diverse types of bioresorbable optical sensors. Device designs adapted from conventional, nonresorbable optical sensor technologies, such as Fabry-Pérot interferometer (FPI) sensors, with sensitivities, accuracies, and measurement ranges that meet various clinical needs, are of particular interest.

A major challenge in the development of bioresorbable sensors as implantable monitors with clinical-grade performance is in realizing consistent mechanical and electrical/optical behavior while immersed in biofluids throughout the clinically relevant monitoring periods, before ultimately undergoing complete bioresorption. Most bioresorbable pressure and temperature sensors operate stably in simulated biofluids for only a few days, which is insufficient for many envisioned applications, such as in intracranial monitoring during a recovery period following traumatic brain injury (*15*, *16*). In other cases, the stability remains to be studied thoroughly (*23*–*25*). The limitations arise mainly from the inability of bioresorbable encapsulation layers, including polymers such as silk fibroin (*22*) and poly(lactic-*co*-glycolic acid) (PLGA) (*20*) and inorganic layers such as silicon dioxide (*26*, *27*) and various metal oxides (*28*) formed by chemical or physical vapor deposition, to prevent permeation of water into the active regions of the devices for extended periods. Recent work demonstrates that ultrathin layers of silicon dioxide thermally grown on device-grade silicon wafers (t-SiO_2_) can be used as bioresorbable encapsulation layers, to enable stable operation of intracranial pressure (ICP) and temperature (ICT) sensors over a period of 25 days in rats (*17*).

Here, we demonstrate two types of bioresorbable optical pressure sensors that build on and leverage some of these ideas. One uses an FPI design, and the other exploits photonic crystal (PC) structures. Both systems rely on pressure-induced deflections of Si NM diaphragms and the resulting changes in the thickness of an air cavity or in the lattice parameters of a PC, both of which cause shifts in resonant peak positions in the reflection spectra. These platforms can also be configured to sense temperature in a manner that relies on the temperature-dependent refractive index of silicon. Fabrication procedures based on wafer bonding of silicon-on-insulator (SOI) wafers followed by back-etching of the handle wafers enable device structures encapsulated in thin layers of t-SiO_2_, with stable operating times of over a week. Both PLGA optical fibers and free-space detection setups serve as bioresorbable optical interfaces. In vitro dissolution studies and histopathological evaluations confirm the biodegradability of these complete systems. Acute measurements of ICP and ICT in rats suggest some potential for clinical application. The results not only establish routes to pressure and temperature sensor technologies that are completely bioresorbable and MRI compatible but also represent generalizable platforms for other classes of bioresorbable optical sensors.

## RESULTS

### Bioresorbable FPI sensors

**Fig. 1:**
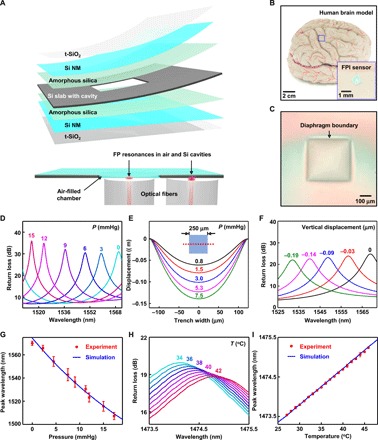
Materials and designs for bioresorbable FPI-based pressure and temperature sensors. (**A**) Schematic illustration of a bioresorbable FPI pressure and temperature sensor composed of a thermally grown silicon dioxide (t-SiO_2_) encapsulation layer, a Si NM, amorphous silica adhesion layer, and a silicon slab. The layers of t-SiO_2_ and Si NM serve as pressure-sensitive diaphragms that seal an air chamber formed by bonding with a silicon slab with a feature of relief etched onto its surface. The bottom image shows a cross-sectional view of a sensor integrated with two optical fibers that deliver light to diaphragm and nondiaphragm regions of the device, thereby enabling pressure and temperature sensing, respectively. (**B**) Photograph of a device placed on an adult brain model. The inset shows a magnified view. (**C**) Optical micrograph of the top diaphragm. (**D**) Optical spectra collected from a bioresorbable FPI pressure sensor immersed in PBS (pH 7.4) at room temperature under different pressures. The FP resonance peak associated with the air cavity shifts to the blue with increasing pressure. (**E**) 3D-FEA of the vertical displacement of the diaphragm along the midsection (red dotted line) at different pressures. (**F**) CEM simulations of optical spectra obtained at different displacements of the diaphragm. (**G**) Calibration curve for the FPI pressure sensor (red) compared with simulation results (blue) obtained in (C) and (F). (**H**) Optical spectra collected from a bioresorbable FPI temperature sensor immersed in PBS at varying temperatures. The FP resonance peak associated with silicon shifts to the red with increasing temperature. (**I**) Calibration curve for the FPI temperature sensor (red) compared with optical simulation results (blue). Circles and error bars in (G) and (I) indicate means ± SEM for three measurements.

Figure 1A illustrates the materials and device geometries associated with bioresorbable pressure and temperature sensors that use an FPI design, and consist entirely of inorganic materials such as coatings of thermally grown silicon dioxide (t-SiO_2_, ~10 nm), single-crystalline Si NMs (250 nm), adhesion layers of amorphous silica (~200 nm), and a slab of silicon with a square cavity (dimensions, 750 μm by 750 μm by 10 μm; cavity area, 250 μm by 250 μm; see fig. S1 for the step-by-step fabrication process) defined by etching a structure of relief onto its surface (*17*). Bonding layers of t-SiO_2_ and Si NMs onto the top and bottom sides of this slab yield two pressure-sensitive diaphragms that float over a sealed air chamber in between. Optical fibers connected to a tunable laser and photodetector couple light in and out of the device to enable measurement of pressure and temperature via changes in measured reflection spectra. Here, the choices of material and the thickness of the bottom diaphragm, through which light enters and leaves, play important roles in minimizing confounding signals in the pressure sensor output (see note S1 and fig. S2 for details). The layers of t-SiO_2_ serve as water barriers that delay the penetration of biofluids to the Si NMs, thereby defining device lifetimes that can reach several weeks, depending on the designs and requirements. Figure 1B presents an image of a bioresorbable FPI sensor on a human brain model, and Fig. 1C shows an optical micrograph of the diaphragm.

In vitro evaluations in a thermodynamic environment similar to that found inside the body demonstrate the functional capabilities of these types of sensors. Figure S3 (A and B) shows photographs of the setups used to calibrate the pressure and temperature responses. A custom-made test chamber consists of a plastic container installed with rubber fittings that form air and watertight seals around plastic tubes that incorporate one-way valves and connect the chamber to a syringe and commercial pressure sensor for pressure control. Thermoelectric heaters/coolers and thermistors installed in a beaker filled with phosphate-buffered saline (PBS, pH 7.4) provide a simulated biofluid environment with temperature control. Collecting reflection spectra at different pressures and temperatures from FPI sensors immersed in the PBS yields data for calibration.

The sensitivity of the FPI pressure sensor follows from optical resonances associated with the air cavity and reflective interfaces defined by the polished surfaces of the Si NMs above and below. These FP resonance patterns shift in wavelength in response to pressure-induced changes in the geometry of the cavity, according to (*29*)

$$\lambda_{q}=\frac{2\mathrm{nt}}{q}$$ (1)

where λ*_q_* represents the wavelength position of a reflection peak, *n* is the refractive index of the material in the cavity, *t* is the thickness of the cavity, and *q* is the mode number. The rise in pressure reduces the thickness of the cavity, which, in turn, shifts the resonant peaks toward shorter wavelengths, thereby serving as the basis for a measurement of external pressure via spectral analysis. Figure 1D and fig. S3C show representative optical spectra collected from a bioresorbable FPI pressure sensor immersed in PBS at 37°C across a range of pressures relevant to in vivo monitoring.

Mechanical and optical simulations yield insights into the mechanisms associated with these measured responses. Three-dimensional finite element analysis (3D-FEA) yields estimates of the vertical displacement of the diaphragm due to changes in external pressure (Fig. 1E). Computational electromagnetism (CEM) analysis determines spectra for cavity geometries defined by these mechanical computations (Fig. 1F). The combined simulation data match well with experimental pressure calibration curves in Fig. 1G. The results indicate a pressure sensitivity of −3.8 nm/mmHg and an accuracy of ±0.40 mmHg for the FPI pressure sensor in the 0- to 15-mmHg range (see note S2 for the definition). Note that this pressure range can be expanded substantially by either changing the pressure sensor sensitivity or by using a light source with an even broader spectral coverage, or by tracking other resonances, as shown in fig. S3 (C and D). Approaches that rely on calibration tables and automated peak detection algorithms can be used in this case (*30*).

Temperature sensing with this same platform relies on light coupled into noncavity regions of the device, which consist mostly of silicon. Here, the FP resonance peaks shift with temperature because of the positive thermo-optic coefficient of silicon (*31*). Tracking temperature is important for the application of bioresorbable FPI pressure sensors as well, as the changes in surrounding temperature induce volumetric changes of the air inside the cavity, thereby adding a temperature-dependent response to the pressure sensor output (see note S3 and fig. S4 for details). Figure 1H and fig. S5 show representative optical spectra obtained from a bioresorbable FPI temperature sensor during immersion in PBS, tested within a range of physiologically relevant temperatures. A thermistor provides a reference measurement of temperature (setup in fig. S3). The relationship between the resonant wavelength and temperature follows from Eq. 1. A comparison between the in vitro temperature calibration curve and optical simulation data appears in Fig. 1I (see note S4 for calculations). The results indicate a temperature responsivity of 0.090 nm/°C and an absolute accuracy of ±0.12°C for temperatures between 27° and 46°C, sufficient for applications of interest here.

### Bioresorbable PC microcavity sensors

**Fig. 2:**
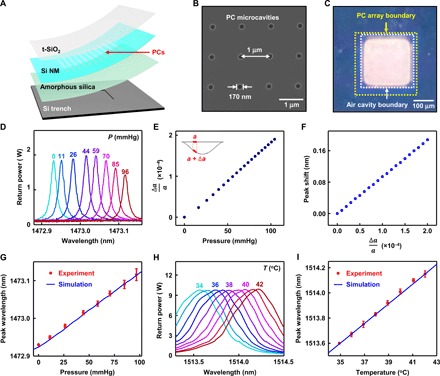
Materials and designs for bioresorbable PC microcavity-based pressure and temperature sensors. (**A**) Schematic illustration of a bioresorbable PC microcavity pressure and temperature sensor, based on a PC structure fabricated in a Si NM, which, together with layers of t-SiO_2_ for encapsulation, constitutes the diaphragm. The diaphragm seals an air cavity formed by bonding to a silicon substrate with a feature of relief etched into its surface. A layer of amorphous silica serves as an adhesion layer. A free-space detection setup couples light to the top surface of the device. Light delivered to the diaphragm and nondiaphragm regions of the device enables pressure and temperature sensing, respectively. (**B**) SEM image of PC structure (*r* = 85 nm, *a* = 1 μm) fabricated in the Si device layer of an SOI wafer by electron beam lithography. (**C**) Image of the diaphragm region of the device, illustrating the alignment of the PC cavity array (300 μm by 300 μm; yellow box) in the diaphragm over the trench etched on the silicon substrate (250 μm by 250 μm; white box). (**D**) Optical spectra collected from the PC pressure sensor placed in air at room temperature under different pressures. The PC resonance peak shifts to the red with increasing pressure. (**E**) 3D-FEA simulation of the average lattice constant (over the area of a 150-μm-diameter circle) at different pressures. (**F**) CEM simulation of the shift in the peak wavelength induced by changes in the lattice constant. (**G**) Calibration curve for the bioresorbable PC pressure sensor (red) compared with simulation data (blue) obtained in (E) and (F). (**H**) Optical spectra obtained from the PC temperature sensor in air at different temperatures. (**I**) Calibration curve for the bioresorbable PC temperature sensor (red) compared with optical simulation data (blue). Circles and error bars in (G) and (I) indicate means ± SEM for three measurements.

Figure 2 illustrates a bioresorbable optical pressure and temperature sensor system based on PC structures (*32*–*36*) formed on a flexible diaphragm that forms one side of an airtight chamber. These sensors yield sharp resonance peaks with a high Q factor (in the range of 10^3^ to 10^5^) that enables high measurement precision; their nonrepeating resonance signals also eliminate the issues of mode overlap inherent to FPI sensors (see note S5 for details) (*37*).

Figure 2A shows materials and designs for bioresorbable sensors based on this type of design, with Si NMs (thickness, ~250 nm) that have nanoscale arrays of holes (area, 300 μm by 300 μm), t-SiO_2_ encapsulation layers (~3 μm), adhesion layers of amorphous silica (~200 nm), and thin silicon substrates with square trenches defined into their surfaces (substrate, 1 mm by 1 mm by 15 μm; trench, 250 μm by 250 μm by 10 μm; see fig. S6 and note S6 for details on the fabrication process). A free-space detection setup delivers light to the PC structure to allow spectroscopic measurements of reflectivity that can be correlated to pressure and temperature, following details described in the next paragraph. Figure 2B shows a scanning electron microscope (SEM) image of the PC structure (holes with radii of 85 nm and a lattice constant of 1 μm, in 2D square lattice) fabricated in the top silicon device layer of an SOI (top Si, ~250 nm; buried SiO_2,_ ~3 μm) wafer (step 2 in fig. S6). Figure 2C shows an optical image of the device, illustrating the alignment of the PC on the diaphragm (yellow box, area: 300 μm by 300 μm) above the square trench on the silicon substrate (white box, area: 250 μm by 250 μm).

Pressure and temperature calibration of this type of sensors in air relies on a setup (fig. S7D) that consists of an airtight chamber with glass walls to allow optical access, mounted on a stage with position and angle control. An interface to a syringe and commercial pressure sensor via plastic tubes with one-way valves enables pressure control and measurement, respectively. The sensor relies on the collective expansion or shrinkage of the lattice constant of the PC and associated shifts in the resonant peaks in the reflection spectra, as a result of deformations due to changes in external pressure. Solutions to Maxwell’s equations and photonic band diagram theories define a positive, linear correlation between the lattice constant of the PC and its resonant wavelength (refer to section 2.1 on Maxwell’s equations) (*38*). The representative optical spectra shown in Fig. 2D and fig. S8A demonstrate this trend, as an increase in pressure increases the PC lattice constant as a result of bending-induced strains (the thin Si NM lies below the thick t-SiO_2_ in the diaphragm), which, in turn, leads to a red shifting of the resonant peak.

Mechanical simulation using 3D-FEA yields the average lattice constants of the PC structure in the center region of the diaphragm, within a circular area of diameter ~150 μm (corresponding to the size of the beam used for testing), at different pressures (Fig. 2E). CEM defines the optical spectra for a range of lattice constants (Fig. 2F). The combined simulation results match the experimental pressure calibration data (Fig. 2G), which indicate a pressure sensitivity of 1.9 pm/mmHg and an accuracy of ±0.64 mmHg. Here, the sensitivity remains consistent throughout a wide range of pressures (0 to 100 mmHg) because of the nonrepeating resonances of the PC structure.

As with the FPI sensors, PC temperature sensors rely on the temperature-dependent refractive index of silicon for temperature sensing, but their responses depend on solutions to Maxwell’s equations and electromagnetic wave theory rather than the resonating conditions of ray optics (*38*). The theories predict a linear scaling between the resonant wavelength and the refractive index of silicon. Monitoring the optical spectra of light coupled to flat, nondiaphragm regions of the device at different temperatures, controlled by a thermoelectric heater/cooler installed on a sample stage of the pressure calibration test setup, allows for calibration of the sensor response. Figure 2H and fig. S8B show representative optical spectra collected at a range of clinically relevant temperatures. The temperature calibration curve and optical simulation results shown in Fig. 2I indicate a sensitivity of 81 pm/°C and an accuracy within ±0.10°C throughout the clinically relevant temperature range (35° to 42°C).

### Bioresorbable optical interfaces

**Fig. 3:**
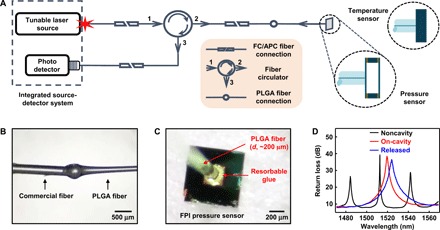
Bioresorbable interfaces to the optical sensors. (**A**) Schematic illustration of the setup used for spectral analysis of bioresorbable FPI sensors. FC, ferrule connector; APC, angled physical contact. (**B**) Photograph of a PLGA fiber [diameter (*d*), ~200 μm] formed at the tip of a commercial SMF. (**C**) Photograph of a PLGA fiber aligned and fixed to the bottom diaphragm of a bioresorbable FPI pressure sensor. The bioresorbable glue holds the parts together. (**D**) FP resonance signals collected at different stages of the process of integrating the fiber with the surface of the sensor. The steps involve aligning the fiber tip to a non–air cavity region of the device (noncavity; black) at a perpendicular incidence and minimizing the distance between the tip and the device, laterally repositioning the fiber to align the tip close to the center region of the diaphragm, applying and curing the bioresorbable glue (on-cavity and glued; red), releasing the device from the water-soluble crystal bond by heating the carrier wafer (released; blue), and removing the residual crystal bond by dipping in warm water.

Bioresorbable optical interfaces between external source-detector systems and sensors deployed inside the body are critical components of the overall systems (*39*–*43*). Figure 3 summarizes an approach based on direct coupling of light through a bioresorbable PLGA fiber, bonded on one end to an FPI sensor and on the other to a conventional single-mode fiber (SMF) that connects to the source and detector. Figure S7 demonstrates a free-space alternative.

Figure 3A outlines the complete FPI sensor setup, including an integrated tunable laser source–photodetector system, a fiber circulator, a series of SMFs, a PLGA fiber, and the sensor. Dipping the tip of SMF (diameter, ~250 μm) into molten PLGA, then pulling it out at a controlled rate, yields the desired fiber dimensions (diameter, ~200 μm) and naturally forms an efficient optical connection between the conventional and bioresorbable fibers (Fig. 3B). Figure S8 (C and D) illustrates the mechanical flexibility (*40*) and propagation loss, respectively, associated with the PLGA fiber. The transmission spectrum of the single mode–to–PLGA composite fiber indicates a low level of noise (±0.1 dB) and modal interference (fig. S8E). The PLGA fiber bonds to the FPI sensor with a bioresorbable skin adhesive (Fig. 3C) (*44*).

Interconnecting the PLGA fiber with the FPI sensor bonded to a temporary carrier wafer (steps 8 to 10 in fig. S1) follows a carefully controlled set of procedures, with measurements of the optical spectra collected after each step (Fig. 3D). The process begins with perpendicular alignment of the fiber tip as close as possible to the flat noncavity surface of the sensor. The measured FP resonances in this configuration correspond mainly to those of the 10-μm-thick silicon substrate (noncavity, black). Lateral displacement of the fiber aligns the fiber tip to the center region of the bottom diaphragm, where the optical spectrum shows a resonant peak associated with the 10-μm-thick layer of air (on-cavity, red). Applying several drops of bioresorbable glue, followed by small amounts of isopropyl alcohol to cure the glue, and releasing the sensor from the temporary carrier wafer completes the process (released, blue; see Materials and Methods for detailed procedures). Figure S8F illustrates the magnitude of noise in the reflection spectrum of the integrated system induced by the modal mismatch between the SMF and the PLGA fiber (see note S7 for details).

Figure S7 illustrates a free-space detection setup used for collecting all data from the PC-based sensors (Fig. 2) (*45*). Optical communication relies on light from either a tunable laser source (sweep range, 1470 to 1570 nm) or a superluminescent diode (SLD, 1440 to 1620 nm) that travels through a circulator and various other optical elements (fig. S7, A to C) before reaching the PC array, which is placed inside a custom test chamber (fig. S7D; described in the previous section). The beam focuses on a circular region (diameter, ~150 μm) of the PC (250 μm by 250 μm), thereby allowing collection of resonance signals that arise from light interactions within the circle, as visualized by an infrared camera (fig. S7, E to G).

### In vitro dissolution studies

**Fig. 4:**
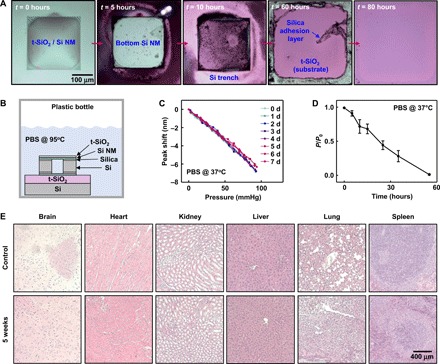
Kinetics of dissolution and histological studies of bioresorbable optical sensors and fibers. (**A**) Optical micrographs at various stages of accelerated dissolution of a bioresorbable FPI sensor due to immersion in PBS (pH 7.4) at 95°C. (**B**) Schematic illustration of the setup used for the dissolution experiment in (A). (**C**) Pressure calibration curves obtained from a bioresorbable FPI pressure sensor (t-SiO_2_ thickness, 1 μm; air chamber thickness, 100 μm), integrated with a commercial optical fiber, soaked in PBS at 37°C for 8 days. The minimal changes in pressure sensitivity provide evidence for the effectiveness of t-SiO_2_ as a biofluid barrier. (**D**) Loss of transmission efficiency of the PLGA fiber over time due to immersion in PBS at 37°C. Circles and error bars indicate means ± SEM for four measurements. (**E**) Representative histological results of the brain, heart, kidney, liver, lung, and spleen organs explanted from a control mouse and a mouse with a bioresorbable FPI sensor implanted in the brain for 5 weeks. The results indicate an absence of abnormalities such as necrosis or inflammation.

The uniqueness of these optical sensor systems is their ability to dissolve completely into biocompatible end products when immersed in aqueous solutions such as the cerebrospinal fluid (CSF) and PBS. Previous reports establish the dissolution kinetics of Si and SiO_2_ in various simulated biofluids at physiological temperatures and values of pH, as well as the biocompatibility of the end product silicic acid [Si(OH)_4_] (*15*, *17*, *21*, *27*). Figure 4A presents optical images of a bioresorbable FPI sensor, composed entirely of Si and SiO_2_, at various stages of accelerated dissolution in PBS at 95°C. Here, the fabrication of a device on a silicon wafer with a layer of thermally grown SiO_2_ prevents penetration of water from the bottom side, to facilitate observation of layer-by-layer dissolution from the top surface of the device (Fig. 4B). The results indicate complete hydrolysis of device layers within 80 hours. An estimation based on Arrhenius scaling and reported activation energies for each material (*46*, *47*) suggests a corresponding time scale of ~195 days at physiological temperatures (~37°C). Optical images of a PLGA fiber immersed in PBS at 37°C, collected over a period of 3 weeks, illustrate the biodegradability of PLGA (fig. S9A) (*15*, *48*).

The t-SiO_2_ encapsulation layer plays a critical role in preventing the premature permeation of water into active device regions, thereby greatly increasing the stable operating lifetime of the device. Pressure calibration curves of an FPI pressure sensor (t-SiO_2_ thickness, 300 nm; air cavity thickness, 100 μm; SMF interconnection) collected over a period of 8 days of immersion in PBS at 37°C reveal highly stable pressure responses throughout the test period (within ±6% variation; Fig. 4C), while those obtained from a device without a t-SiO_2_ layer (air cavity thickness, 10 μm) under otherwise similar test conditions exhibit gradual increases in sensitivity and baseline over time (fig. S9, A and B). This trend results from the thinning of the highly doped Si NM due to hydrolysis, which increases its flexibility and, therefore, the vertical displacement of the diaphragm due to surrounding pressure. The resonant peak of an unprotected bioresorbable PC device (illustrated in Fig. 2B) also gradually red shifts over time while placed in PBS at 65°C. Here, dissolution of the Si NM reduces the thickness and increases the radius of the holes in the PC structure, both of which lead to reduction of the resonant wavelength (*38*), as illustrated by the measurements of peak wavelength shift and Si NM thickness over time (fig. S9, D and E).

Figure 4D shows measurements of transmission efficiency of a PLGA fiber immersed in PBS at 37°C as a function of time. The efficiency decreases notably in ~3 days, because of uptake of water into the polymer, before bioresorption. Figure 4E shows histology results from brain, heart, kidney, liver, lung, and spleen tissues collected from a mouse with an FPI device implanted in the brain after 5 weeks, compared with those from a control mouse without the implant. Histopathological evaluation of the images reveals no sign of inflammation, necrosis, or structural abnormality in any organ.

### In vivo demonstrations in rats

**Fig. 5:**
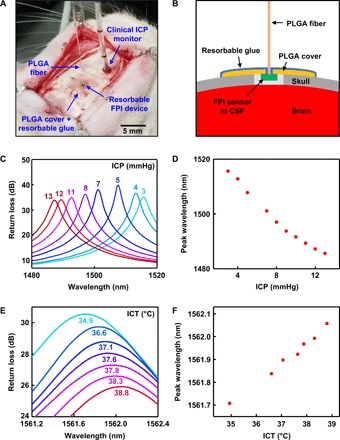
Acute monitoring of ICT and ICP in rats. (**A**) Photograph of a bioresorbable FPI sensor implanted in the intracranial space of a rat for monitoring ICT and ICP. A thin film of PLGA, with a hole in the middle for the fiber, and bioresorbable glue seal the cranial defect to form an airtight environment for accurate pressure sensing. A clinical fiber-optic ICP monitor, implanted nearby, provides reference data. (**B**) Cross-sectional schematic illustration of the device setup during animal testing. Cutting through the dura and exposing the device to CSF allows assessment of the ICP. (**C** and **D**) Optical spectra and pressure calibration curve obtained from a bioresorbable FPI pressure sensor, calibrated against the clinical ICP monitor. Contracting the flank of the rat temporarily raises the ICP by up to ~10 mmHg from the initial level. (**E** and **F**) Optical spectra and temperature calibration curve obtained from a bioresorbable FPI temperature sensor, calibrated against a commercial thermistor implanted nearby. The electrical heating blanket wrapped around the body of the rat gradually increased the ICT. (Photo credit for part A: Jiho Shin, University of Illinois at Urbana-Champaign.)

Acute measurements of ICP and ICT in rats provide data that suggest clinical applicability of bioresorbable FPI sensors. Figure 5 (A and B) illustrates the surgical procedures for implanting the devices into the intracranial space. The process involves drilling a small defect in the skull, implanting the sensor inside, placing a thin film of PLGA with a hole in the middle (dimensions, 5 mm by 5 mm by 10 μm; hole diameter, ~400 μm) on top, and bonding the PLGA film with the skull by applying a layer of bioresorbable glue to form an airtight seal over the intracranial cavity (*44*). The hardened glue helps prevent changes in the sensor-fiber alignment due to shear or slanting effects during and after implantation. Implanting a clinical fiber-optic ICP monitor and/or a commercial thermistor in a separate, nearby defect allows comparison of measurements obtained by bioresorbable and nonresorbable sensors. The sweeping speed of the source-detector system limits the data sampling rate of this system to ~1 Hz.

Squeezing and holding the flank of the rat increases the intra-abdominal pressure, which, in turn, increases the ICP, allowing control over a range of 3 to 13 mmHg depending on the force applied. These ICPs fall within the 0- to 15-mmHg window within which the bioresorbable FPI pressure sensor functions without involving additional modes, thereby simplifying direct comparison between the responses of bioresorbable and commercial devices. Optical spectra and calibration data appear in Fig. 5 (C and D). The pressure sensitivity (−3.1 nm/mmHg) differs from that obtained from in vitro testing because two different optical resonance modes are used in these two cases (the FP resonance peak here has a shorter wavelength).

Wrapping the rat’s body in an electrical heating blanket slowly raises the ICT, thereby allowing comparisons of ICT measured using a bioresorbable FPI temperature sensor and a commercial thermistor over a range of physiological temperatures (near 37°C). The optical spectra and calibration data in Fig. 5 (E and F) indicate a linear temperature sensitivity of 0.089 nm/°C, which is close to the value obtained from in vitro tests. The observed resonant peak corresponds to mode 4 in the full optical spectrum shown in fig. S5. Note S8 and fig. S10 describe the degree of pressure and temperature cross-talk on the pressure sensor response during in vivo measurement.

## DISCUSSION

The materials, device designs, and fabrication approaches introduced here serve as foundations for bioresorbable optical sensor implants that can accurately monitor pressure and temperature inside the body over clinically relevant time frames for various applications, and then naturally disappear via processes of bioresorption to eliminate the need for surgical extraction. The results include two types of optical sensors based on FPI and PC microcavity structures, as well as two optical communication approaches based on single mode–to–PLGA composite fibers and free-space detection setups. Long-term in vitro studies demonstrate the effectiveness of thermally grown layers of SiO_2_ as encapsulation layers in extending the lifetimes of the devices, while histological studies using mouse models establish the biocompatibility of the dissolution products. In vivo monitoring of ICT and ICP in rats demonstrates the potential clinical utility of these systems.

With respect to practical utility as implantable sensor platforms, FPI sensors integrated with optical fibers have a major advantage over PC sensors. The reflection spectrum of a PC sensor with free-space optical communication depends largely on the light incidence angle and position of the beam on the PC, both of which are subject to continuous variation in vivo due to natural movement of the body (e.g., breathing and heartbeat), which introduces noise and complicates data collection. Moreover, any layer of biofluid (e.g., blood and CSF) that coats the PC will absorb incident light and cause loss of light intensity, leading to poor sensitivity and accuracy. Fiber-integrated FPI sensors can avoid both issues because of the immobilized sensor-fiber interface.

Regarding clinical safety, however, the tethering of FPI sensors to an external source/detector system can be problematic, as it restricts the movement of the subject throughout the monitoring period, which may lead to other safety hazards. The opening in the skin and the blood-brain barrier (for intracranial implants), through which the fiber penetrates, also increases the risk of bacterial infection. PC sensors, with free-space detection, have the potential to solve both problems, as they can function wirelessly, and the skin/barrier can be closed.

The device concepts and materials introduced here may enable bioresorbable optical sensor platforms capable of monitoring various other biologically relevant quantities including blood flow, motion, ionic concentrations, respiratory, and heart rates, as important alternatives to electrical sensing approaches.

## MATERIALS AND METHODS

### Fabrication of bioresorbable FPI sensors

Figure S1 shows schematic illustrations of the fabrication process. Mechanical back grinding (Syagrus Systems, USA) reduced the thickness of the silicon handle wafer of an SOI wafer (top Si ~10 μm, buried SiO_2_ ~600 nm, Si wafer ~90 μm; University Wafers, USA) before device fabrication. Photolithography and deep reactive ion etching (STS Pegasus ICP-DRIE) formed a 5 × 5 array of square trenches (250 μm by 250 μm by 10 μm) on the top Si layer. ICP-DRIE and wet etching in hydrofluoric acid (HF, Transene Company Inc., USA) removed the Si wafer and the buried SiO_2_ layer to release a 10-μm-thick slab of Si with trenches on its surface. Solid-state diffusion of phosphorus at 950°C formed doped Si NMs on a separate SOI wafer (SOI-B, top Si ~250 nm, buried SiO_2_ ~3 μm, Si wafer ~97 μm after mechanical grinding; SOITEC, France). Photolithography and ICP-DRIE defined an array of vent holes (area, 100 μm by 100 μm) through the thickness of SOI-B. The wafer bonding process involved spin-coating a layer of diluted poly(dimethylsiloxane) (PDMS, part A/part B/hexane = 10:1:100 by weight, Sylgard 184; Dow Corning, USA) to a thickness of 3 μm on two SOI-B wafer samples, partially curing the PDMS at 110°C for 1 min, transferring the silicon trench on top of one SOI-B, transferring the other SOI-B on top upside down, pressing the wafers together in a steel vise (Toomaker’s vise; Tormach Inc., USA), and fully curing the PDMS by placing the vise in a 70°C convection oven for 2 hours. Heating the vise in a furnace, raising the temperature gradually to 550°C for 2 hours, and maintaining this temperature for an additional 2 hours converted the PDMS to amorphous silica. Next, ICP-DRIE removed the top Si wafer of the bonded sample to expose the buried oxide. Wet etching in buffered oxide etchant (BOE, 6:1; Transene Company Inc., USA) thinned the top buried SiO_2_ layer to a thickness of ~10 nm. Profilometer (Alpha Step D-500; KLA Tencor, USA) measurements confirmed the thickness of the buried oxide. Additional wet etching, as needed, yielded the desired thickness. Photolithography and dry etching (Plasma-Therm RIE) through a stack of buried SiO_2_, Si NM, amorphous silica, and silicon layers isolated individual devices (area, 750 μm by 750 μm). Bonding the sample upside down on a temporary Si wafer using a water-soluble crystal bond (Structure Probe Inc., USA), followed by ICP-DRIE and wet etching in BOE to thin the buried SiO_2_ yielded bioresorbable FPI sensors ready for integration with optical fiber.

### Fabrication and evaluation of biopolymer fibers

Fabrication began by placing a bulk sample of PLGA (lactide:glycolide, 75:25; molecular weight, 66,000 to 107,000; Sigma-Aldrich, USA) into a small glass reservoir and heating to 200°C on a hotplate. Inserting a SMF (model SM980-5.8-125; Thorlabs Inc., USA) with a 250-μm diameter and slowly pulling it vertically yielded a PLGA fiber with a diameter of ~200 μm drawn from the reservoir. The cut-back method enabled measurements of propagation loss for the PLGA fiber. Specifically, coupling PLGA fibers (200 μm in diameter and 4 cm in length) individually to a 633-nm wavelength light-emitting diode (LED) light source (power fixed at 0.5 mW) and measuring the output intensities from the other end of the fiber using a photometer yielded values corresponding to 4-cm-length fibers. Five cycles of cutting off short pieces of fiber, with measurements as described above for each cycle, determined the propagation loss of the PLGA fiber.

### Integration of biopolymer fiber with bioresorbable FPI sensors

A collection of stages and positioners **(**ULTRAlign Precision Fiber Optic Alignment Stages and Positioners; Newport, USA) facilitated alignment of the PLGA fiber, formed at the tip of a commercial SMF, to an FPI-based pressure and temperature sensor bonded to a temporary carrier wafer by the water-soluble crystal bond (Structure Probe Inc., USA). An optical spectrum analyzer (model AQ6370B; Yokogawa Test & Measurement Corporation, Japan) captured the FP resonances throughout the integration process to ensure a proper, perpendicular orientation, and a minimal gap between the fiber tip and the device. The process involved aligning the fiber tip to a nondiaphragm region of the FP device, laterally repositioning the tip onto the center of diaphragm, and then applying small drops of bioresorbable glue (TissueSeal, USA) followed by introducing isopropyl alcohol to cure the glue, heating the carrier wafer using a thermoelectric heater (CUI Global Inc., USA) to melt the crystal bond, detaching the device and dipping it into warm water to remove the residual crystal bond.

### Calibration of pressure and temperature responses of bioresorbable FPI sensors

Figure S3 (A and B) show the in vitro test setup used for calibrating the pressure and temperature responses of the bioresorbable FPI sensors. The setup included an airtight test chamber (built from a plastic container fitted with rubber rings) that contained a beaker filled with PBS (pH 7.4, 0.01 M; Sigma-Aldrich, USA), in which the FPI sensor and a commercial thermistor (DigiKey, USA) were immersed. A thermoelectric heater/cooler installed underneath the platform enabled temperature control. Specifically, a thermoelectric controller (model TC-720; TE Technology Inc., USA), connected with the thermistor and thermoelectric heater/cooler, located outside the chamber maintained the desired temperature of the PBS via automated proportional-integral-differential feedback control of the temperature. Plastic tube connections to a plastic syringe through a one-way valve (Plastic Double Head Check Valve; Tasharina Corp., USA) and a commercial pressure sensor (NeuLog, USA) allowed for control and measurement of the pressure inside the chamber, respectively. An integrated tunable laser source–photodetector (8163B Lightwave Multimeter; Keysight Technologies Inc., USA) system connected with a circulator (model 6015-3-APC; Thorlabs Inc., USA) enabled optical communication with FPI sensors. Collecting optical spectra at different pressures by pumping air into the chamber using the syringe allowed calibration of the pressure response. Calibration of the temperature response involved obtaining optical spectra at different temperatures, controlled by the thermoelectric control system, while leaving the chamber open to atmospheric pressure.

### Fabrication of PC microcavity sensors

Schematic illustrations of the fabrication steps appear in fig. S6. The process involved fabrication of the PC microcavity array (radius, 85 nm; lattice constant, 1 μm; total area, 300 μm by 300 μm) on the top Si of SOI-B by electron beam lithography. Photolithography and ICP-DRIE defined alignment marks through the thickness of the SOI-B wafer, visible on the bottom surface of the wafer. Photolithography and ICP-DRIE defined a square trench (250 μm by 250 μm by 5 μm) on the top Si of SOI-A. Wafer bonding of SOI-A (containing the trench) and SOI-B (containing the PC structure) followed the same procedures used for fabrication of the FPI sensors. Alignment marks etched through the SOI-A and on SOI-B wafers allowed proper positioning of the PC cavity array over the trench. Next, ICP-DRIE removed the top Si wafer (SOI-B) of the bonded sample to expose the buried oxide. Photolithography, DRIE, and wet etching in BOE isolated individual devices (area, 1 mm by 1 mm). Spin-coating a layer of photoresist on the top surface, etching the Si wafer and buried oxide of SOI-B by ICP-DRIE and wet etching in BOE, and removing the photoresist by dry etching (March RIE) completed the fabrication.

### Calibration of pressure and temperature responses of bioresorbable PC sensors

Figure S7 illustrates the free-space detection setup used for calibration of the PC sensors in air. Incident light from either a tunable laser source or an SLD (model S5FC1550P-A2; Thorlabs Inc., USA) traveled through a circulator and other optical elements before finally passing through the glass window of a test chamber (Waterproof Housing for GoPro Camera; FitStill, USA) connected to a syringe and a commercial pressure sensor through plastic tubes installed with check valves. Optimization of the PC reflection signal involved visualizing the position of the beam using an infrared camera, monitoring the signal amplitude using the optical spectrum analyzer, and moving the XYZ stage and tilting the sample-holding plate to maximize the output. Figure S7G shows an infrared image of the beam focused on the center region of the PC pressure sensor diaphragm. Acquiring reflection spectra at various pressures yielded calibration data. Calibration of the PC temperature sensor relied on the same alignment and testing methods, but with a thermoelectric heater/cooler installed on the sample holder.

### Mechanical and optical simulations

3D-FEA determined the vertical displacement of the diaphragm (FPI sensor; Fig. 1E) and the average change in the PC lattice constant (PC sensor; Fig. 2E) at different pressures. Abaqus software simulated the diaphragm as a quadrilateral shell element (S4R) with clamped boundaries.

Stanford S^4^ optical simulation tools yielded the spectrum profiles at different maximum deformation depths (FPI sensor; Fig. 1F) and photonic lattice constants (PC sensor; Fig. 2F), using the rigorous coupled-wave analysis method. For optical simulation of bioresorbable temperature sensor responses, the S^4^ package also accounted for optical dispersion relations of silicon (*49*) and silicon dioxide (*50*), as well as the thermal expansion properties of each material (*51*).

### In vitro dissolution experiments

Immersing a bioresorbable FPI sensor in 50 ml of PBS maintained at 95°C for a fixed time, rinsing with deionized water, drying, and inspecting under an optical microscope yielded images of the accelerated dissolution process. A plastic bottle with a screw cap, installed with a digital thermometer probe penetrating through the cap, served as the test chamber.

Placing a PC structure formed on an SOI-B wafer in 0.4 M PBS at 65°C for several minutes, rinsing, drying, and collecting optical spectra using the free-space detection method allowed measurement of resonant shifts in response to thinning of the silicon. SEM images revealed the changes in Si thickness based on the increase in average radius of the holes in the PC induced by dissolution.

For measurements of changes in optical transmission properties of the PLGA fiber during biodegradation, a small reservoir made of PDMS allowed exposure of small lengths of fiber to PBS, leaving the two ends for coupling to a 633-nm LED light source (power, 0.5 mW) and connecting to a photometer. The system was maintained at 37°C using a temperature controller. Measuring the output intensity from the photometer over time yielded recordings of the changes in optical transmission during biodegradation of PLGA.

### Histopathological evaluation

The Institutional Animal Care and Use Committee (IACUC) of Northwestern University approved the procedures. Ultraviolet (UV) radiation sterilized the bioresorbable FPI sensors overnight. Surgical procedures involved anesthetizing female CD-1 mice (Charles River Laboratories, USA) using isoflurane gas, cutting open the scalp to expose the skull, immobilizing the head using a stereotaxic frame, opening a small defect on the skull by drilling, implanting the sensor inside, sealing the intracranial cavity with a bioresorbable tissue adhesive (TissueSeal, USA), and suturing the skin. Histopathological analysis followed from euthanasia of two mice after 5 weeks and extraction of the liver, spleen, heart, kidney, brain, and lung tissues.

### Evaluation in animal models

All procedures of the animal study followed recommendations in the Guide for the Care and Use of Laboratory Animals of the National Institutes of Health. The IACUC of Washington University in St. Louis (protocol number 20170189) approved the protocol. Male Lewis rats weighing 250 to 350 g (Charles River Laboratories, USA) received subcutaneous injections of buprenorphine hydrochloride (0.05 mg kg^−1^; Reckitt Benckiser Healthcare Ltd., USA) for pain management and of ampicillin (50 mg kg^−1^; Sage Pharmaceuticals, USA) to prevent infection at the implantation site before the surgery. The implantation procedures involved anaesthetizing the rat with isoflurane gas, holding the head in a stereotaxic frame, opening a craniectomy defect and dura by drilling, implanting the bioresorbable device on the cortical surface, and sealing the craniectomy with a PLGA sheet (~10 μm thick; with a hole in the middle for fiber connection) and bioresorbable glue (TissueSeal, USA). A clinical ICP monitor (Camino System, model MPM-1; Integra LifeSciences, USA) or a commercial thermistor (DigiKey, USA) implanted in a nearby craniectomy enabled comparison testing to demonstrate the accuracy of the pressure and temperature measurements by the bioresorbable sensor, respectively. Squeezing and holding the rat’s body induced increments in ICP. Wrapping the body with electrical heating blanket gradually raised the ICT.

## Supplementary Material

http://advances.sciencemag.org/cgi/content/full/5/7/eaaw1899/DC1

Download PDF

## SUPPLEMENTARY MATERIALS

Supplementary material for this article is available at http://advances.sciencemag.org/cgi/content/full/5/7/eaaw1899/DC1 Note S1. Design considerations for FP pressure sensor. Note S2. Definition and calculation of device sensitivity and accuracy. Note S3. Temperature dependence of pressure sensor response. Note S4. Calculation of FPI temperature sensor sensitivity. Note S5. Comparison of reflection spectra of FPI and PC sensors. Note S6. Scalability and yield of bioresorbable PC sensor fabrication process. Note S7. Modal mismatch and noise in single mode–to–PLGA composite fiber. Note S8. Pressure and temperature cross-talk during in vivo measurement. Fig. S1. Schematic illustrations of steps for fabricating bioresorbable FPI pressure and temperature sensors. Fig. S2. Calculations of free spectral ranges and design optimization of bioresorbable FPI pressure sensors. Fig. S3. In vitro setup for calibrating the response of the bioresorbable FPI pressure sensor. Fig. S4. Effects of temperature on the response of a bioresorbable FPI pressure sensor. Fig. S5. Full temperature calibration curves for a bioresorbable FPI temperature sensor. Fig. S6. Schematic illustrations of fabrication procedures for bioresorbable PC cavity-based pressure and temperature sensors. Fig. S7. Free-space detection setup for testing bioresorbable PC-based pressure and temperature sensors. Fig. S8. Optical properties of bioresorbable PC sensors and PLGA fiber. Fig. S9. In vitro dissolution of a bioresorbable optical sensor. Fig. S10. Simultaneous recordings of a rat’s ICP and ICT during flank contract/release experiment using reference pressure and temperature sensors.
