# Supplementary material for: Bioresorbable optical sensor systems for monitoring of intracranial pressure and temperature
Source: Sci Adv. 2019 Jul 5;5(7):eaaw1899. doi: 10.1126/sciadv.aaw1899 (PMC6611687; doi:10.1126/sciadv.aaw1899)
Supplement: http://advances.sciencemag.org/cgi/content/full/5/7/eaaw1899/DC1 [file supp_5_7_eaaw1899__index.html]

Science Advances | Science AdvancesAAASSearchScience AdvancesMenu

## Supplementary Materials

**This PDF file includes:**

- Note S1. Design considerations for FP pressure sensor.
- Note S2. Definition and calculation of device sensitivity and accuracy.
- Note S3. Temperature dependence of pressure sensor response.
- Note S4. Calculation of FPI temperature sensor sensitivity.
- Note S5. Comparison of reflection spectra of FPI and PC sensors.
- Note S6. Scalability and yield of bioresorbable PC sensor fabrication process.
- Note S7. Modal mismatch and noise in single mode–to–PLGA composite fiber.
- Note S8. Pressure and temperature cross-talk during in vivo measurement.
- Fig. S1. Schematic illustrations of steps for fabricating bioresorbable FPI pressure and temperature sensors.
- Fig. S2. Calculations of free spectral ranges and design optimization of bioresorbable FPI pressure sensors.
- Fig. S3. In vitro setup for calibrating the response of the bioresorbable FPI pressure sensor.
- Fig. S4. Effects of temperature on the response of a bioresorbable FPI pressure sensor.
- Fig. S5. Full temperature calibration curves for a bioresorbable FPI temperature sensor.
- Fig. S6. Schematic illustrations of fabrication procedures for bioresorbable PC cavity-based pressure and temperature sensors.
- Fig. S7. Free-space detection setup for testing bioresorbable PC-based pressure and temperature sensors.
- Fig. S8. Optical properties of bioresorbable PC sensors and PLGA fiber.
- Fig. S9. In vitro dissolution of a bioresorbable optical sensor.
- Fig. S10. Simultaneous recordings of a rat’s ICP and ICT during flank contract/release experiment using reference pressure and temperature sensors.

Download PDF

**Files in this Data Supplement:**

- Adobe PDF - aaw1899\_SM.pdf
