# Supplementary material for: Bioresorbable optical sensor systems for monitoring of intracranial pressure and temperature
Source: Sci Adv. 2019 Jul 5;5(7):eaaw1899. doi: 10.1126/sciadv.aaw1899 (PMC6611687; doi:10.1126/sciadv.aaw1899)
Supplement: Download PDF [file aaw1899_SM.pdf]

Science Advances
AAAS

advances.sciencemag.org/cgi/content/full/5/7/eaaw1899/DC1

Supplementary Materials for

Bioresorbable optical sensor systems for monitoring of intracranial pressure and temperature

Jiho Shin, Zhonghe Liu, Wubin Bai, Yonghao Liu, Ying Yan, Yeguang Xue, Irawati Kandela, Maryam Pezhouh, Matthew R. MacEwan, Yonggang Huang, Wilson Z. Ray, Weidong Zhou*, John A. Rogers*

*Corresponding author. Email: jrogers@northwestern.edu (J.A.R.); wzhou@uta.edu (W.Z.)

Published 5 July 2019, *Sci. Adv.* **5**, eaaw1899 (2019)
DOI: 10.1126/sciadv.aaw1899

This PDF file includes:

Note S1. Design considerations for FP pressure sensor.
Note S2. Definition and calculation of device sensitivity and accuracy.
Note S3. Temperature dependence of pressure sensor response.
Note S4. Calculation of FPI temperature sensor sensitivity.
Note S5. Comparison of reflection spectra of FPI and PC sensors.
Note S6. Scalability and yield of bioresorbable PC sensor fabrication process.
Note S7. Modal mismatch and noise in single mode–to–PLGA composite fiber.
Note S8. Pressure and temperature cross-talk during in vivo measurement.
Fig. S1. Schematic illustrations of steps for fabricating bioresorbable FPI pressure and temperature sensors.
Fig. S2. Calculations of free spectral ranges and design optimization of bioresorbable FPI pressure sensors.
Fig. S3. In vitro setup for calibrating the response of the bioresorbable FPI pressure sensor.
Fig. S4. Effects of temperature on the response of a bioresorbable FPI pressure sensor.
Fig. S5. Full temperature calibration curves for a bioresorbable FPI temperature sensor.
Fig. S6. Schematic illustrations of fabrication procedures for bioresorbable PC cavity-based pressure and temperature sensors.
Fig. S7. Free-space detection setup for testing bioresorbable PC-based pressure and temperature sensors.
Fig. S8. Optical properties of bioresorbable PC sensors and PLGA fiber.
Fig. S9. In vitro dissolution of a bioresorbable optical sensor.
Fig. S10. Simultaneous recordings of a rat's ICP and ICT during flank contract/release experiment using reference pressure and temperature sensors.

Note S1. Design considerations for FP pressure sensor.
The free spectral range (FSR) of a Fabry-Pérot interferometer (FPI), defined as the distance between adjacent resonant wavelengths in the FP interference spectrum, can be estimated by the following relationship

$$(\Delta\lambda_0)_{fsr} \approx \frac{\lambda_0^2}{2nd} \quad (0.1)$$

where λ_0 is the resonant wavelength, *n* the refractive index, and *d* the thickness of the layer. The FSRs of constituent materials of a bioresorbable FPI sensor, as functions of individual layer thickness, appear in fig. S2A.

The reflection from an FPI that includes multiple layers contains FP resonances that correspond to each layer. Therefore, to avoid overlap of multiple signals and obtain FP signal from a specific layer, it is necessary to differentiate the FSRs of the layers. For a bioresorbable FPI pressure sensor with 10 μm-thick air cavity, the thickness of the silicon support (portion of silicon substrate that lies below air cavity) must be optimized to differentiate the silicon FSR from that of air. Figure S2B estimates Si to air FSR ratio of 0.5 for a silicon support of 5 μm thickness, which results in FP resonance signal that contains mixture of silicon and air peaks (fig. S2C, black). A thinner silicon support of 0.25 μm thickness leads to a ratio of 11.5, and hence a sharp FP resonance peak (blue). More precisely, the 5 μm Si resonance signal will not appear in the target wavelength window, making it easier to distinguish the resonant wavelength.

Note S2. Definition and calculation of device sensitivity and accuracy.
Recording the resonant wavelengths every ~3 mmHg (or 1 ^oC) from 3 sensors placed in the same calibration chamber, calculating (1) the average and (2) standard deviation of peak shift (in units of nm) at each pressure/temperature, and dividing (1) by pressure/temperature yields the device sensitivity (in units of nm/mmHg or nm/^oC). Taking the absolute values of (2), taking the average of this value for all pressure/temperature, and dividing it by device sensitivity yields the sensor accuracy (in mmHg or ^oC).

Note S3. Temperature dependence of pressure sensor response.
The thermodynamic relationship between pressure, volume, and temperature makes the influence of pressure and temperature on the volume of air (more specifically, the thickness of the air cavity) inside an FPI pressure sensor intrinsically inseparable. As an example, figs. S4A and D show pressure calibration curves obtained at different temperatures using bioresorbable FPI pressure sensors with air cavity thicknesses of 10 μm and 100 μm, respectively. The 10 μm and 100 μm air cavity devices have pressure sensitivities of -3.4 nm/mmHg and -0.81 nm/mmHg (independent of temperature; figs. S4B and E) and temperature sensitivities of 13 nm/^oC and 3.9 nm/^oC (figs. S4C and F), respectively. Their relative sensitivities to pressure over temperature, estimated as each device's pressure sensitivity divided by its temperature sensitivity, are -0.26 ^oC/mmHg and -0.21 ^oC/mmHg, which are similar in magnitude. The results indicate the difficulty of de-convoluting the dependence of pressure sensor response on pressure and temperature by modifying the sensor design; it also suggests the need for separate transducers for monitoring pressure and temperature, to enable de-convolution of the effects post-measurement.

Note S4. Calculation of FPI temperature sensor sensitivity.
A simple analytical equation to estimate the temperature sensitivity of an FPI temperature sensor can be obtained by taking the derivative of Equation (1)

$$\frac{d\lambda_q}{dT} = \frac{dn}{dT}\frac{2t}{q} + \frac{dt}{dT}\frac{2n}{q} = \frac{dn}{dT}\frac{\lambda_q}{n} + \alpha\lambda_q$$

Here, $d\lambda_q/dT$ represents the temperature sensitivity, dn/dT, the thermo-optic coefficient of Si, λ_q, the resonant wavelength, n, the refractive index of Si, and α, the thermal expansion coefficient of Si. Inserting $dn/dT = 1.88 \times 10^{-4}$, $\lambda_q = 1550$ nm, $n = 3.47$, and $\alpha = 3.3 \times 10^{-6}$ yields an expected temperature sensitivity of 0.089 nm/K, which is close to the experimental results.

Optical simulation data shown in Fig. 1I provides a more precise estimation, as it relies on solution to wave equations using Stanford Stratified Structure Solver, S^4 (*52*). To simulate the reflection spectra obtained from an FPI temperature sensor at different temperatures, the software requires constructing the device layer structure, inserting the temperature dependent permittivity of each material, and scanning the excitation wavelength.

Note S5. Comparison of reflection spectra of FPI and PC sensors.
The reflection spectrum of the bioresorbable PC pressure sensor contains a single dominant resonant peak (figs. S8A and B), achieved by tuning the hole radius and lattice constant of the PC structure. The resonant peak shifts linearly in response to pressure change, and there exists a one-to-one match between the resonant wavelength and external pressure.

In contrast, the reflection spectrum of the FPI pressure sensor appears periodic and self-repeating (fig. S3C). There are multiple modes (peaks) that can be tracked to determine the pressure. Tracking a single resonant peak, however, can create confusion when the spectrum shifts more than one period of the FP resonance, as the resonant wavelength of one mode at the new pressure can overlap with that of another mode at the base pressure.

A mathematical demonstration looks as follows

$$\lambda_q = \frac{2nt_0}{q}$$

Equation above defines λ_q as the resonant wavelength of mode q in the reflection spectrum of an FPI pressure sensor that has air cavity thickness of t_0. Assuming an increase in external pressure causes the air cavity thickness to decrease to $t_0 - \lambda_q/2n$, the resonant wavelength of a nearby mode $q-1$ is

$$\lambda_{q-1} = \frac{2n(t_0 - \frac{\lambda_q}{2n})}{q-1} = \frac{2nt_0 - \frac{2nt_0}{q}}{q-1} = \frac{\frac{2nt_0}{q}(q-1)}{q-1} = \frac{2nt_0}{q} = \lambda_q$$

This shows that the resonant wavelengths of two modes can overlap at different pressures. Details appear in “Multi-Beam Interference” section in Chapter 9 of reference (*37*).

Note S6. Scalability and yield of bioresorbable PC sensor fabrication process.
All fabrication steps for PC sensors are likely scalable, as they consist of processes such as spin-coating, photolithography, wafer bonding, and silicon etching, which are carried out in wafer-scale in the industry. E-beam lithography process used to fabricate PC cavity array (step 2) may be an exception, and a more scalable method such as interference lithography or nanoimprint lithography may help improve the fabrication speed and throughput.

The overall fabrication yield for the PC sensor is around 80% during small-scale tests using ~1 cm x 1 cm wafer pieces. Wafer bonding, PDMS calcination, and Si handle wafer etching steps (steps 4-6) appear to have the lowest yield. Optimization of the spin-coated PDMS film thickness, vent hole size/separation, and DRIE etch rate/time may help improve the yield.

Note S7. Modal mismatch and noise in single mode–to–PLGA composite fiber.
Figure S8F shows the reflection spectra collected from three FPI pressure sensors integrated with a commercial SMF, single mode-to-5 mm PLGA composite fiber, and single mode-to-20 mm PLGA composite fiber. Optical spectra collected through composite fibers show greater noise due to the modal mismatch between SMF and PLGA fiber. Nonetheless, the magnitude of noise in the reflection loss for single mode-to-5 mm PLGA fiber is within ±0.3 dB, much smaller than the amplitude of the resonant peak (10-25 dB). While quantifying the error in reading out the resonant wavelength (x-axis) introduced by this noise (y-axis) can be challenging, the low noise level suggests that the effect should be very small, almost negligible.

Note S8. Pressure and temperature cross-talk during in vivo measurement.
Figure S10 shows recordings of a rat's ICP and ICT collected throughout a flank contract/release test using pressure and temperature sensors implanted in a rat's intracranial cavity. Contracting the rat's body raises the ICP and ICT by ~7 mmHg and ~0.1 ^{o}C, which correspond to -24 nm and 1.3 nm shift (figs. S4A and B) in the FPI pressure sensor response, respectively. The results indicate that the degree of cross-talk is relatively insignificant during a typical in vivo measurement.

Supplementary Figures

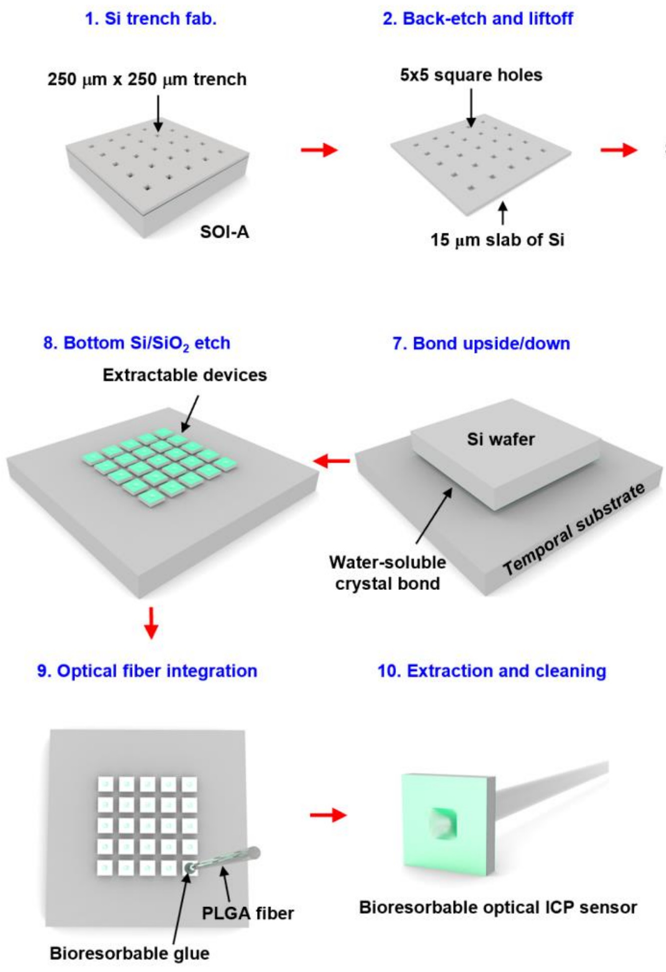

Fig. S1. Schematic illustrations of steps for fabricating bioresorbable FPI pressure and temperature sensors. (1) Fabrication of an array of trenches on the silicon substrate of a silicon-on-insulator (SOI-A) wafer by photolithography and inductively-coupled plasma-deep reactive ion etching (ICP-DRIE) process, (2) back-etching of the silicon handle wafer (thinned to ~100 μm by mechanical grinding) by ICP-DRIE and liftoff by wet etching the buried oxide layer in hydrofluoric acid (HF) to release the Si device layer with holes (Si trench), (3) wafer bonding of two SOI-B wafers using a spin-coated layer of PDMS, (4) curing of the PDMS at 70°C for 2 hours, followed by calcination in a furnace at 550°C for 2 hours to convert to amorphous silica, (5) etching of the handle wafers and buried oxide layers in the top SOI-B, (6) isolating individual FPI sensors by patterned etching of the Si NM and buried SiO_2 using reactive ion etching (RIE) and wet etching in buffered oxide etchant (BOE), respectively, (7) bonding of the sample upside-down on a temporary silicon wafer using water-soluble crystal bond after heating the wafer above the melting temperature of crystal bond, (8) integrating the PLGA fiber using a bioresorbable glue, and (9) releasing the device from the wafer by heating, followed by removal of the residual crystal bond by dipping in warm water.

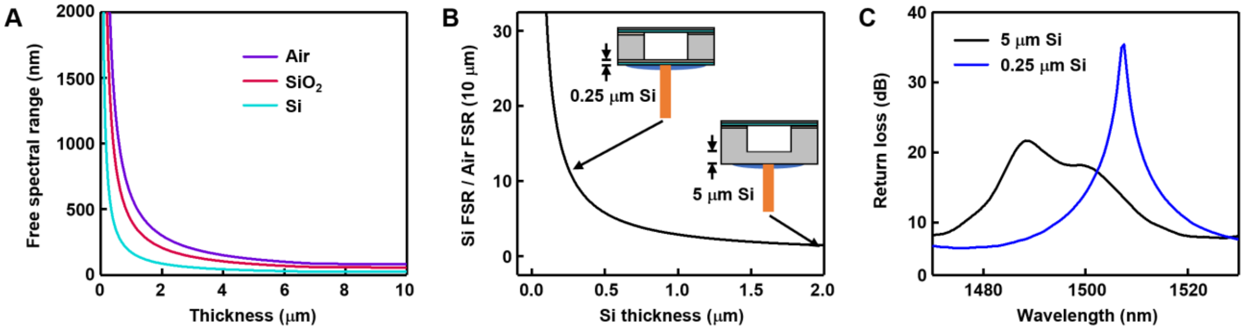

Fig. S2. Calculations of free spectral ranges and design optimization of bioresorbable FPI pressure sensors. (**A**) Estimations of FSRs of Si, SiO_2, and air as functions of layer thicknesses. (**B**) Ratio of FSRs of Si to 10 µm-thick air. The schematic illustrations and arrows indicate two sensor designs with different thicknesses of the silicon support and the corresponding FSR ratio. (**C**) Optical spectra collected from the two device designs in (**B**).

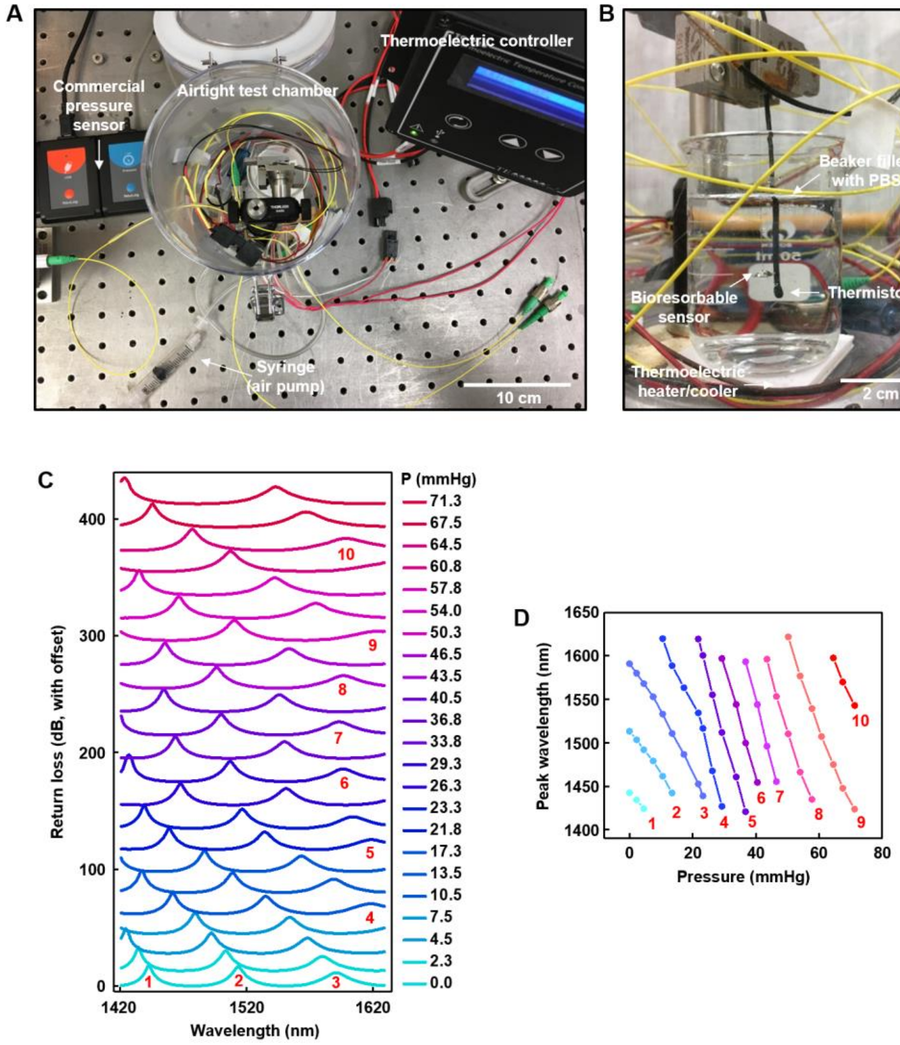

Fig. S3. In vitro setup for calibrating the response of the bioresorbable FPI pressure sensor. (**A, B**) Photograph of the test set-up. A custom airtight test chamber contains a beaker filled with phosphate buffered saline (PBS, pH 7.4), in which the device is immersed. A thermoelectric controller, connected to a thermistor and thermoelectric heater/cooler, enables automated feedback control of the temperature of the PBS. Plastic tube connections to a syringe via a one-way valve, together with a commercial pressure sensor enable manual control of the pressure. (**C, D**) Optical spectra and pressure calibration curve obtained from a bioresorbable FPI pressure sensor soaked in PBS at room temperature, in the pressure range of 0-70 mmHg, illustrating the optical shift in response to changes in pressure. Red numbers indicate all FP resonance peaks (modes) observed over this spectral range. The difference in pressure sensitivities of each mode necessitates tracking of the mode number and wavelength range. (Photo credit for parts A and B: Zhonghe Liu, University of Texas at Arlington).

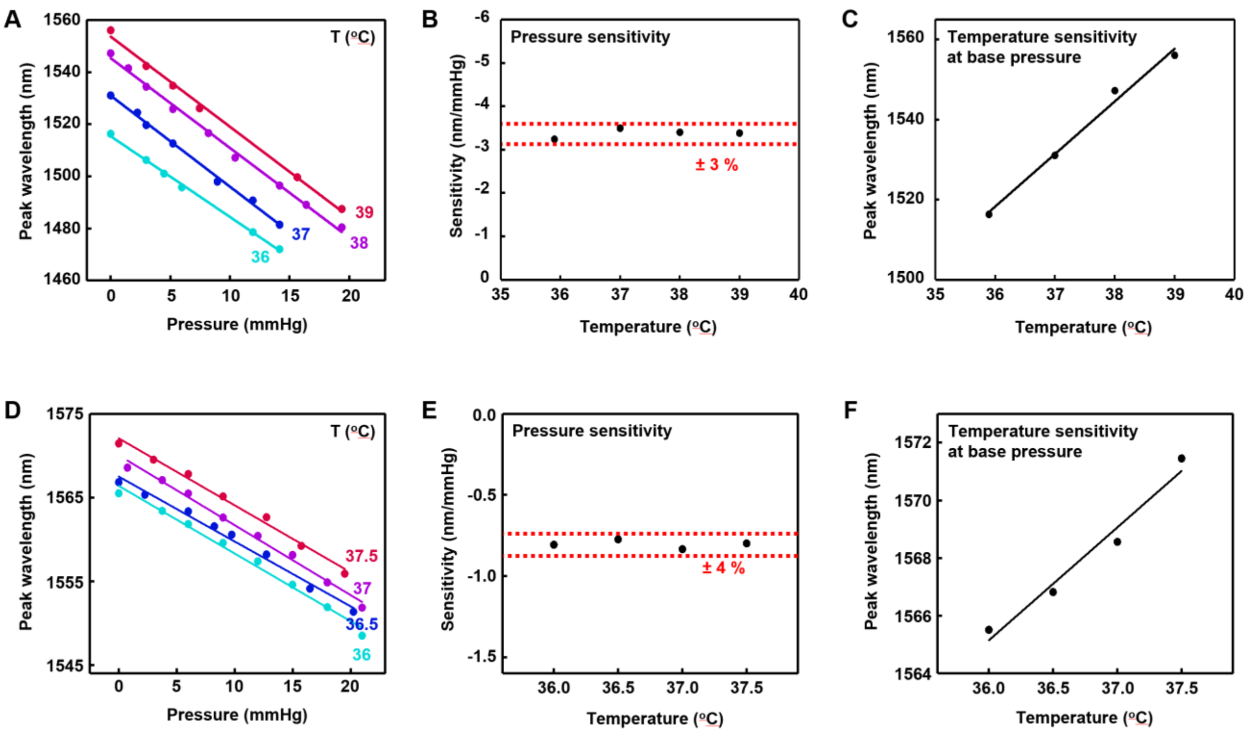

Fig. S4. Effects of temperature on the response of a bioresorbable FPI pressure sensor. (**A**) Pressure calibration curves for an FPI pressure sensor with air cavity thickness of ~10 µm, immersed in PBS that is heated to temperatures relevant to intracranial monitoring. (**B**) Pressure sensitivity of the sensor in **A** at different temperatures. (**C**) Temperature sensitivity of the sensor in **A** under atmospheric pressure. (**D**) Pressure calibration curves for an FPI pressure sensor with air cavity thickness of ~100 µm, immersed in PBS at different temperatures. (**E**) Pressure sensitivity of the sensor in **D** at different temperatures. (**F**) Temperature sensitivity of the sensor in **D** under atmospheric pressure.

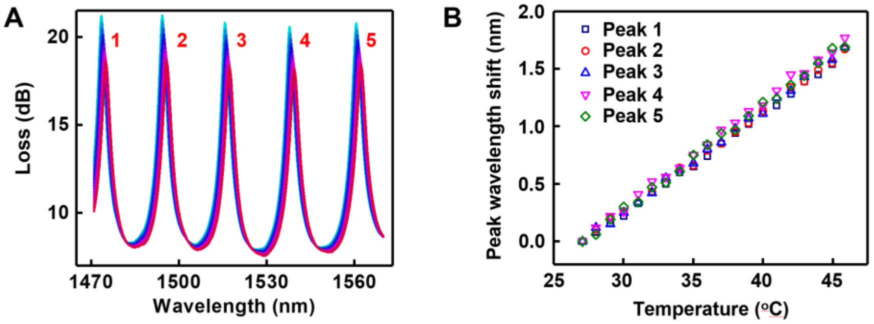

Fig. S5. Full temperature calibration curves for a bioresorbable FPI temperature sensor. (**A**) Optical spectra collected from an FPI temperature sensor immersed in PBS under atmospheric pressure and varying temperatures (27-46 ^{o}C), observed over a wavelength range of 1470-1570 nm. Five modes/peaks are visible over this range. (**B**) Calibration curves of the 5 modes/peaks, plotted in terms of peak wavelength shift.

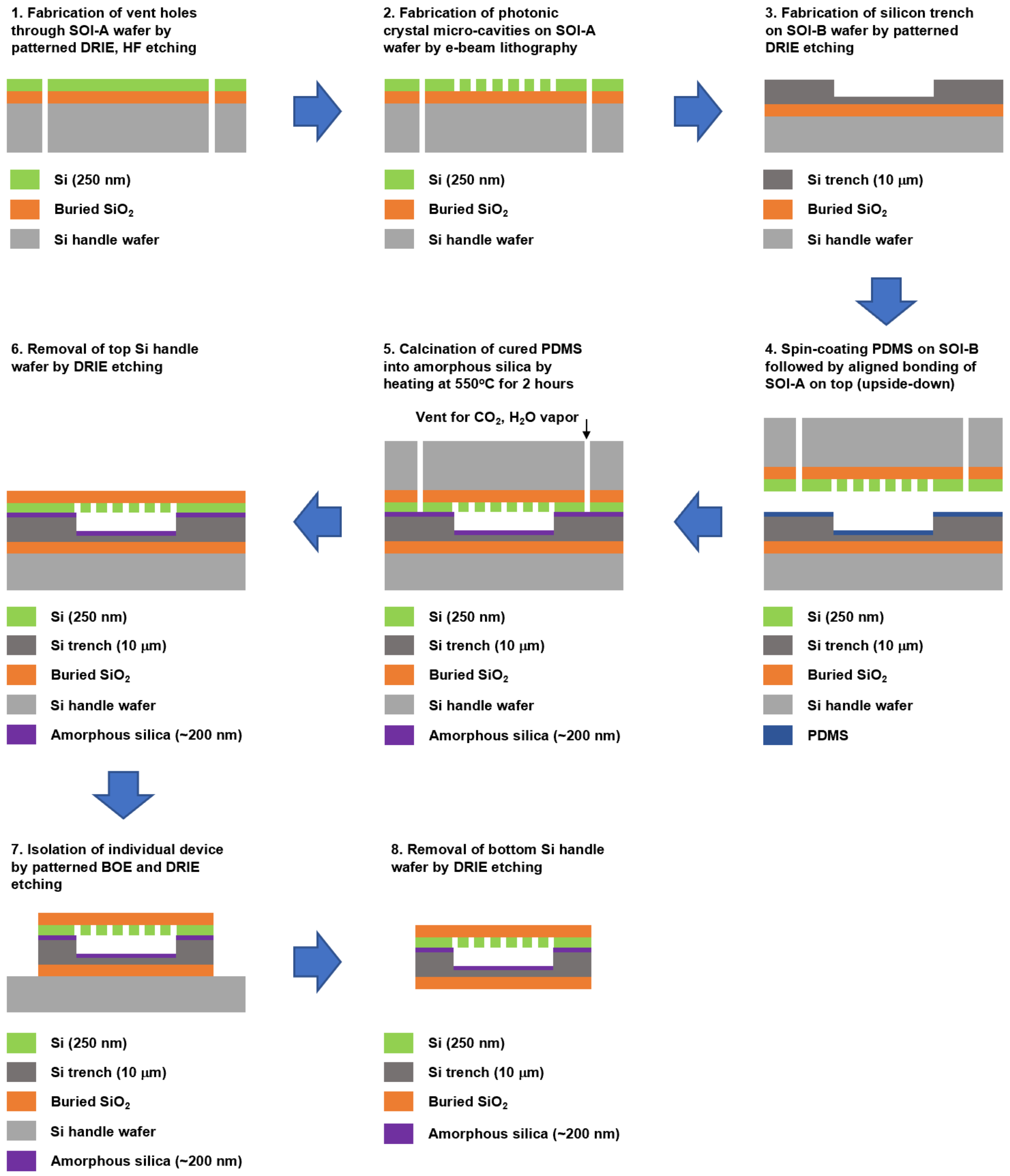

Fig. S6. Schematic illustrations of fabrication procedures for bioresorbable PC cavity-based pressure and temperature sensors. (1) Fabrication of vent holes etched through the SOI-A wafer by photolithography, ICP-DRIE, and HF etching processes. (2) Fabrication of PC structure on the SOI-A wafer by electron-beam lithography. (3) Fabrication of a square trench on the device layer of the SOI-B wafer by photolithography and ICP-DRIE etching. (4) Spin-coating of PDMS on the SOI-B wafer, followed by aligned bonding of the SOI-A wafer, upside-

down, on top to align the PC array over the trench. (5) Calcination of cured PDMS to convert the material to amorphous silica, by heating in a furnace at 550 ^{o}C for 2 hours. (6) Removal of the silicon handle wafer of SOI-A by ICP-DRIE etching. (7) Isolation of individual PC pressure sensors by photolithography, ICP-DRIE, and HF etching. (8) Removal of the silicon handle wafer of SOI-B by ICP DRIE.

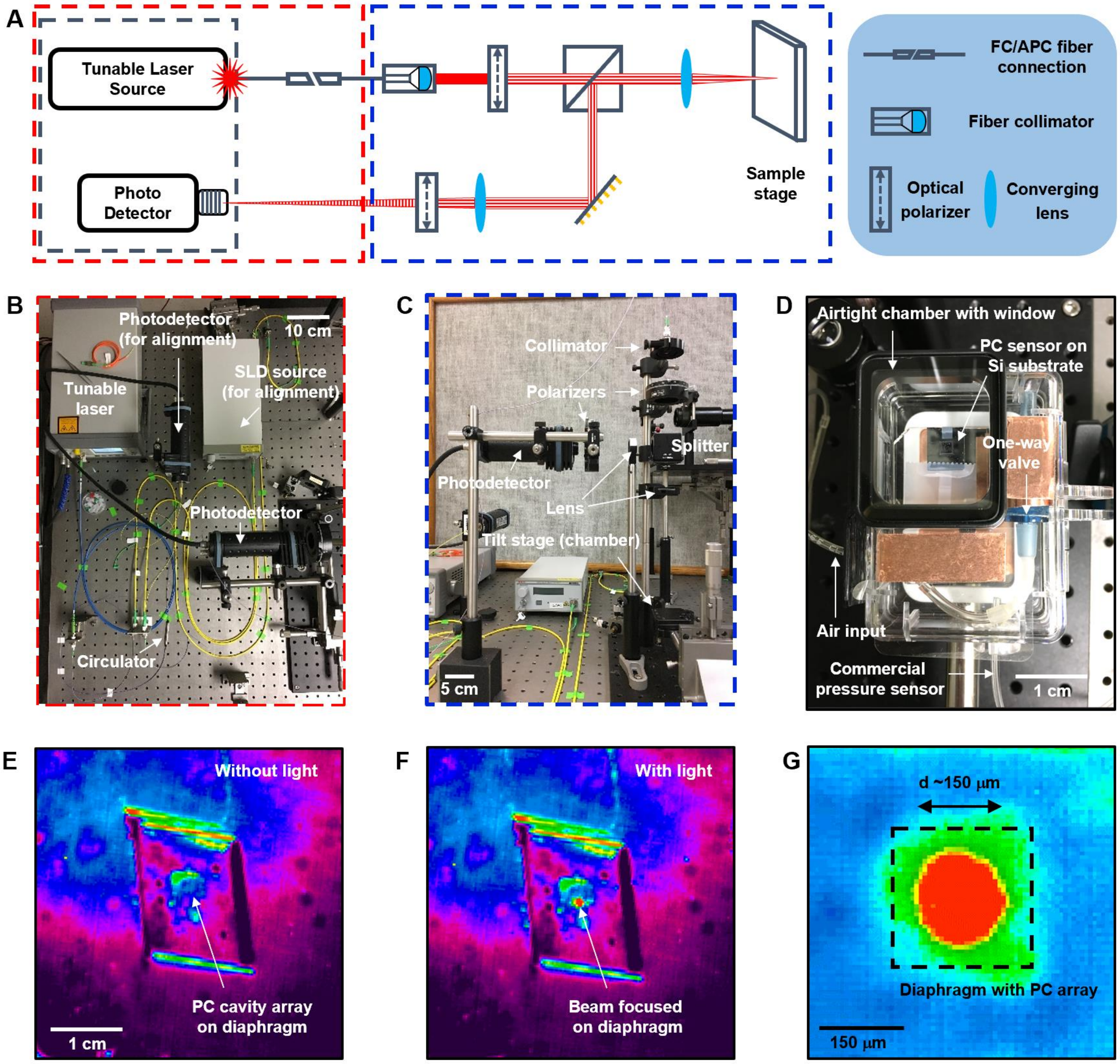

Fig. S7. Free-space detection setup for testing bioresorbable PC-based pressure and temperature sensors. (**A**) Schematic illustration of the set-up. (**B-D**) Photographs of the set-up. The test chamber shown in (**D**) rests on the tilt stage in (**C**), facing vertically upward. Plastic tube connections between the chamber and a syringe, connected to a one-way valve, and a commercial pressure sensor allow calibration of the pressure response of the PC pressure sensor. Light communicates with the bioresorbable PC sensor, mounted on a carrier silicon wafer, through a transparent window of the airtight chamber. (**E-G**) Infrared images of the sensor illustrate the beam (d ~150 μm) aligned to the sensor diaphragm (area: 250 μm x 250 μm), which contains the PC structure. The device areas with and without PC cavity array (area: 300 μm x 300 μm) are difficult to distinguish in the IR images due to their small sizes. (Photo credit for parts B, C and D: Zhonghe Liu, University of Texas at Arlington).

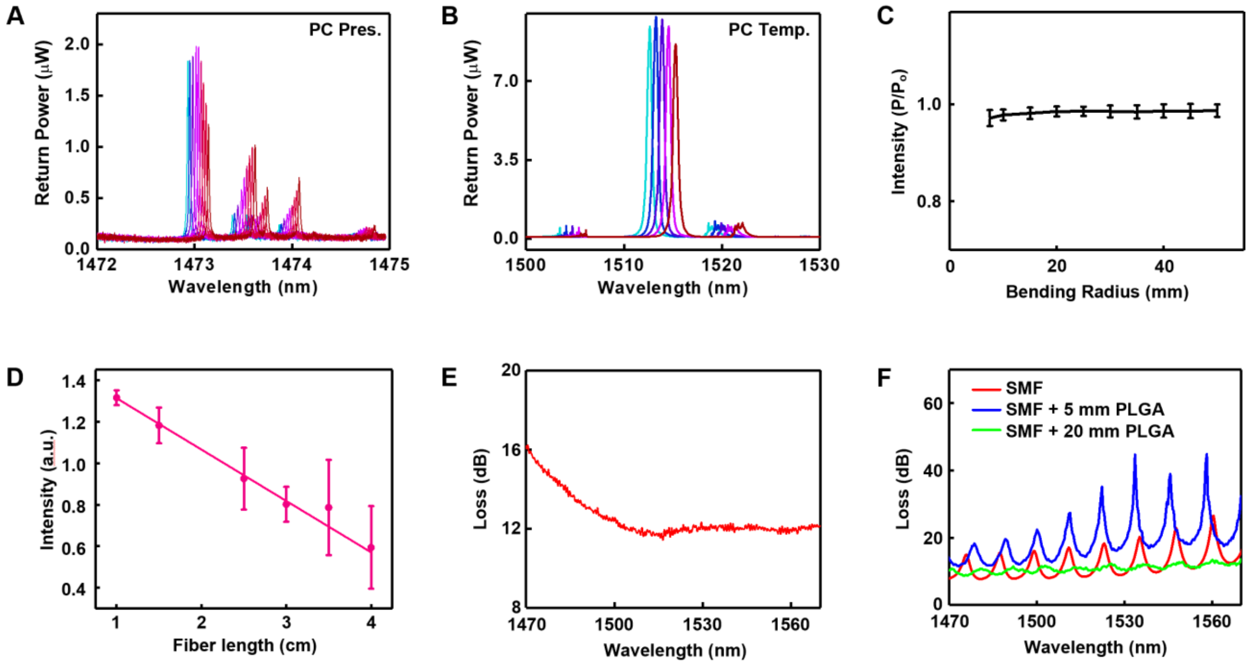

Fig. S8. Optical properties of bioresorbable PC sensors and PLGA fiber. (**A, B**) Optical spectra collected from PC pressure and temperature sensors over the full wavelength range attainable by the tunable laser source (1470-1570 nm). (**C**) Measured transmission intensity of PLGA fiber in air at various bending radii. Bending in the range of 10-40 mm bending radius leads to negligible (within 0.5 %) variation in the transmission intensity. Light wavelength, 633 nm. Diameter of PLGA fiber, 150 μm. P_o: measured transmission intensity when the PLGA fiber is straight. P: measured transmission intensity when the PLGA fiber is bent. (**D**) Propagation loss obtained over a 4 cm length of PLGA fiber (d ~200 μm). The error bars in C and D represent s.d. of 4 measurements. (**E**) Transmission spectrum of single mode-to-5 mm PLGA composite fiber. The noise level is within ±0.1 dB, indicating low level of modal interference. (**F**) Reflection spectra collected from bioresorbable FPI pressure sensors integrated with SMF and single mode-to-PLGA composite fibers with different lengths of PLGA fiber.

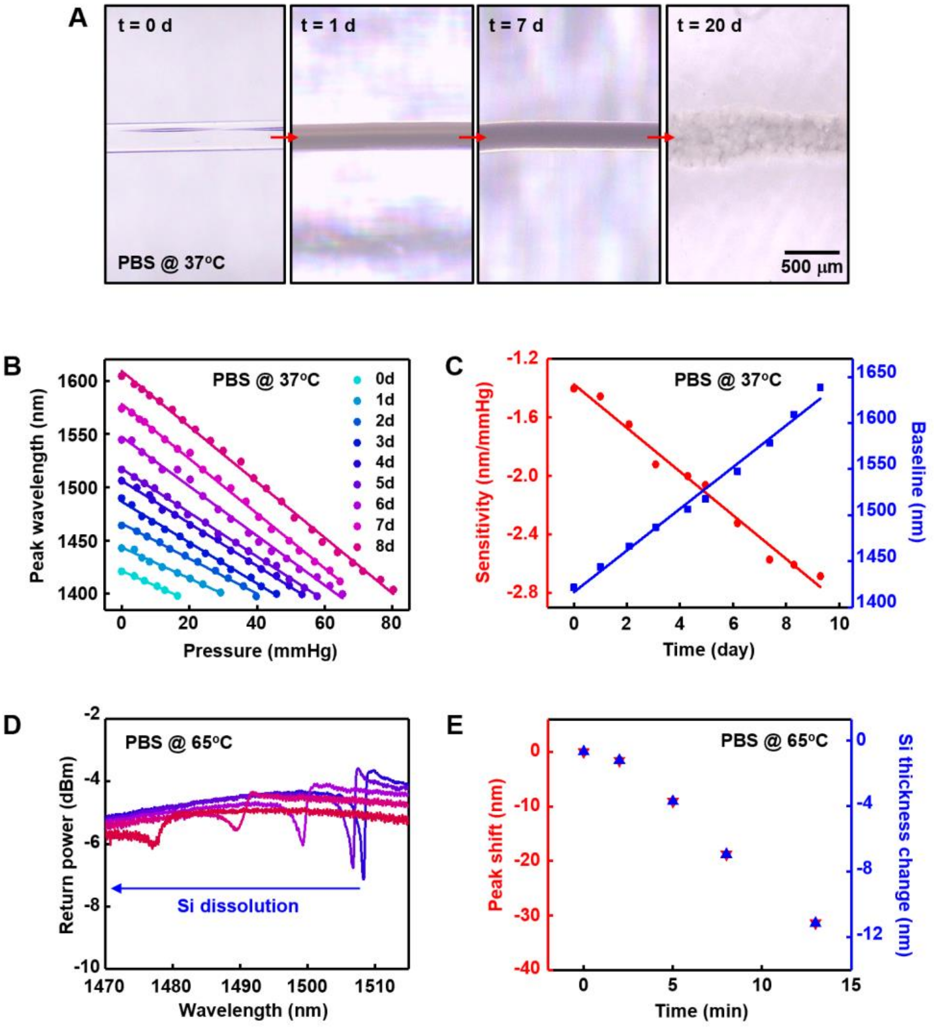

Fig. S9. In vitro dissolution of a bioresorbable optical sensor. (**A**) Images obtained at various stages of dissolution of a PLGA fiber immersed in PBS at 37°C. (**B**) Optical spectra collected from a bioresorbable FPI pressure sensor without a t-SiO_2 encapsulation layer for 8 days of immersion in PBS at 37°C. (**C**) Changes in pressure sensitivity (red) and baseline (blue) as a function of time based on the optical spectra presented in (**B**). (**D**) Optical spectra collected from a PC cavity array formed on the device layer of the SOI-B wafer immersed in 0.4 M PBS at 65°C throughout a 13 minute-monitoring period. The resonant peak wavelength shifts to the blue as the dissolution of Si progresses. (**E**) Peak wavelength shift (red) and change in silicon thickness (blue) over time, based on data in (**D**).

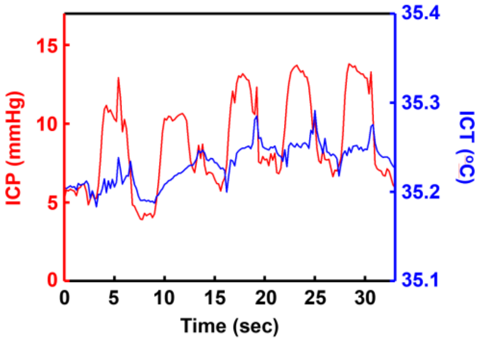

Fig. S10. Simultaneous recordings of a rat's ICP and ICT during flank contract/release experiment using reference pressure and temperature sensors. Variation in ICP and ICT are ~7 mmHg and ~0.1 ^{o}C, respectively, upon each contract/release of the rat's flank.
